# Alveolar soft part sarcoma: a clinicopathological and immunohistochemical analysis of 26 cases emphasizing risk factors and prognosis

**DOI:** 10.1186/s13000-024-01450-z

**Published:** 2024-01-30

**Authors:** Yi Zhang, Yuchen Huang, Yanzi Qin, Ningning Yang, Panpan Yang, Nan Li, Zhenzhong Feng

**Affiliations:** 1 Department of Pathology, Bengbu Medical College, Anhui, China; 2 Department of Pathology, The Second Affiliated Hospital of Anhui Medical University, Anhui, China

**Keywords:** Alveolar soft part sarcoma, Molecular features, Clinicopathological features, TFE3, Prognostic factors

## Abstract

**Objective:**

This study aimed to investigate the clinicopathological features and prognostic indicators of alveolar soft part sarcoma (ASPS).

**Methods:**

The characteristics of 26 ASPS patients diagnosed at our hospital between January 2011 and January 2019 were retrospectively analysed.

**Results:**

The data for 12 male and 14 female patients, with a median age of 27.5 years, were assessed. The clinical symptoms mainly included painless enlarged masses in deep soft tissues. ASPS had a characteristic pathological morphology. Twenty-four patients were positive for TFE3, and *TFE3* gene rearrangement was detected in 12 patients. Among the 26 patients who completed follow-up, 14 had metastasis, 1 had local recurrence, and 7 died. Kaplan–Meier survival analysis revealed that prognosis was significantly correlated with sex, tumour size and metastasis (*P* < 0.05). Multivariate Cox regression analysis revealed that sex and metastasis were independent prognostic risk factors for patients with ASPS (*P* < 0.05).

**Conclusion:**

ASPS is a rare soft tissue sarcoma of unknown origin that occurs in young people, has a slow but metastatic course, and is associated with a poor 5-year survival rate among patients with metastasis. ASPS has character TFE3 protein and gene expression, and the diagnosis is relatively specific. The diagnosis requires comprehensive analysis of clinical history, histological morphology, and immunohistochemistry.

## Introduction

Alveolar soft part sarcoma (ASPS) was first described by Christopherson et al. [1] in 1952. The disease is a soft tissue sarcoma of unknown histological origin [2], accounting for less than 1% of all soft tissue sarcomas [3]. ASPS diagnosis is mainly based on pathological histological observations. In most cases, typical histological and ultrastructural features, which include the presence of cytoplasm containing periodic acid-Schiff (PAS)-positive nonamyloid material, are observed. ASPS has a characteristic chromosomal translocation t(X;17)(p11.2;q25), which forms the ASPSCR1::*TFE3* fusion gene [4]. Due to the rare nature of the disease and its atypical clinical symptoms, ASPS is often misdiagnosed or missed. In this study, we retrospectively analysed the data for 26 ASPS patients, including clinicopathological features, differential diagnoses, prognostic factors, and the literature, to improve the understanding of the disease course and to facilitate treatment planning.

## Materials and methods

### Patient data

Clinicopathological data for 26 ASPS patients diagnosed at our hospital between January 2011 and January 2019 were collected. This study was reviewed and approved by the ethics committee of our hospital. All patients provided written informed consent. The methods of collecting of database information were all in accordance with the guidelines of the Declaration of Helsinki. Among the patients considered, 14 were female and 12 were male, with a median age of 27.5 years (7–68 years). ASPS was primarily located on the extremities (11 patients) or on the trunk or other sites (15 patients). The median diameter of the primary tumour was 5 cm. In total, 26 patients were treated surgically, 2 of whom received postoperative adjuvant radiotherapy (Table 1).

**Table 1:** Clinical information for 26 patients with ASPS

Characteristic		Value
Sex	Male	12 (46.2%)
	Female	14 (53.8%)
Age (years), median (range)		(27.5) 7–68
Tumour size (cm), median (range)		5.0 (2.0–12.0)
Follow-up (months), median (range)		69.5 (9-106)
Tumour location, n (%)	Extremities	11 (42.3%)
	Trunk	10 (38.5%)
	Left neck	1 (3.8%)
	Right oral floor	1 (3.8%)
	Tongue	2 (7.7%)
	Right orbit	1 (3.8%)
Recurrence		1 (3.8%)
Metastasis, n (%)	Lung	7(26.9%)
	Liver	2(7.7%)
	Lung and brain	2(7.7%)
	Lung and liver	1(3.8%)
	Lung and transverse clon	1(3.8%)
	Multiple sites including the lung	1(3.8%)
Necrosis and capsule		1 (3.8%)
Necrosis		5 (19.2%)
Capsule		4 (15.4%)
Necrosis and infiltration		3 (11.5%)
Infiltration		1 (3.8%)

### Methods

Specimens were fixed in 10% neutral formalin, embedded in paraffin, sectioned into 4 μm slices, stained with heamatoxylin and eosin (HE) and immunohistochemically stained. Immunohistochemistry was performed via an EnVision two-step method with the following primary antibodies: tumour cell transcription factor E3 (TFE3), S-100 protein, junctional protein (desmin), cytokeratin (CK), epithelial membrane antigen (EMA), CD68, and smooth muscle actin (SMA) (Maixin Biotechnology Development Co., Ltd., Fuzhou, China). The staining procedure included positive and negative controls and was performed according to the manufacturer’s instructions. In some cases, PAS cytochemical staining was performed. The antibodies used to target the nucleus included TFE3, Ki-67, and S-100. Those with cytoplasmic and/or cell membrane targets included CgA, CK, and EMA.

According to the TFE3 interpretation criteria reported by Sharain et al., the positive percentage of tumor nuclei was initially divided into “negative”, “1+” (10% positive cells), “2+” (10– 50% positive cells) and “3+” (positive cells > 50%). However, only cases with an immunoreactive score of “2+” or “3+” were considered positive [5]. Similarly, nuclear staining intensity in other cases was classified as “negative”, “weak”, “moderate” or “strong”; however, only moderate and strong staining intensity was considered to be positive in keeping with the original scoring system of Argani et al. [6] Immunohistochemical findings were interpreted by two pathologists with associate senior titles or higher. Fluorescence in situ hybridization (FISH) was performed for 12 of the 26 patients, using a *TFE3* (Xp11.2) gene breakage probe assay (Anbiping Pharmaceutical Technology Co., Ltd, Guangzhou, China). All the procedures were performed strictly according to the manufacturer’s instructions.

### Follow-up

Patients were followed-up until November 2019, resulting in a median follow-up duration of 69.5 months (9–106 months) among the study participants. Seven of the 26 patients died —5 (71.4%) males and 2 (28.6%) females. All seven patients died of the disease. Fourteen patients developed postoperative metastases, including 7 to the lung, 2 to the liver, 2 to both the lung and brain, 1 to both the lung and liver, 1 to the lung and transverse colon, and 1 to multiple sites including the lung.

In a 38-year-old male patient, a 12.0 cm tumour was located in the chest wall, with a clear necrotic area at the time of tumour dissection and a diameter of 2.5 cm, rare mitotic figures, and strongly positive TFE3. No metastasis after surgery was observed, and the patient was in a fair overall condition at the final follow-up (45 months postdiagnosis).

In a 56-year-old female patient, the primary site of the 6 cm long tumour was the superior margin of the pancreas. Pathology revealed that the tumour had no necrosis, no infiltration at its border, and few mitotic figures. After resection of the mass and chemotherapy, liver metastasis occurred at postoperative year 5, and recurrence occurred at postoperative year 6. At the time of follow-up (95 months postdiagnosis), the patient was in fair general condition.

A 39-year-old female patient had a 4 cm tumour located in the retroperitoneum, with a peritoneal tumour and rare mitotic figures. No metastasis after surgery was observed, and the patient was in fair general condition at the final follow-up (106 months postdiagnosis).

### Statistical methods

All patients were treated surgically. Tumour size was measured according to the surgical specimen. Survival was calculated as the duration from the date of diagnosis to the date of death or the date of the patient’s final follow-up visit. SPSS 26.0 software was used for statistical analysis, and the Kaplan‒Meier method was used to calculate the survival rate. Prognostic factors were identified via one-way ANOVA. The log-rank test was used to compare the differences between groups, and multivariate Cox regression analysis was used to evaluate relevant prognostic factors, with *P* values of < 0.05 considered to indicate statistical significance.

## Results

### Clinical information

Among the 26 ASPS patients considered, 12 (46.2%) were male, 14 (53.8%) were female, and the age range at diagnosis was 7–68 years, with a median age of 27.5 years. The tumours were located in the lower extremities (9 patients), upper extremities (2 patients), head and neck (5 patients), chest wall (2 patients), back (1 patient), peritoneum (2 patients), iliac fossa (1 patient), groin (2 patients), pancreas (1 patient), and lung (1 patient). All patients were treated surgically. Twelve (46.2%) patients presented with focal disease and 14 (53.8%) had distant metastases, 3 of whom had metastases at the time of diagnosis.

### Pathological features

#### Macroscopic examination

The tumours were primarily located in deep soft tissues, with maximum diameters ranging from 2.0 to 12.0 cm (median diameter, 5.0 cm). Most tumours had clear boundaries and were soft; 5 tumours had capsule, and cut surfaces were greyish white or greyish red. Infiltration and necrosis were observed in 3 patients.

#### Microscopic examination

**Fig. 1:**
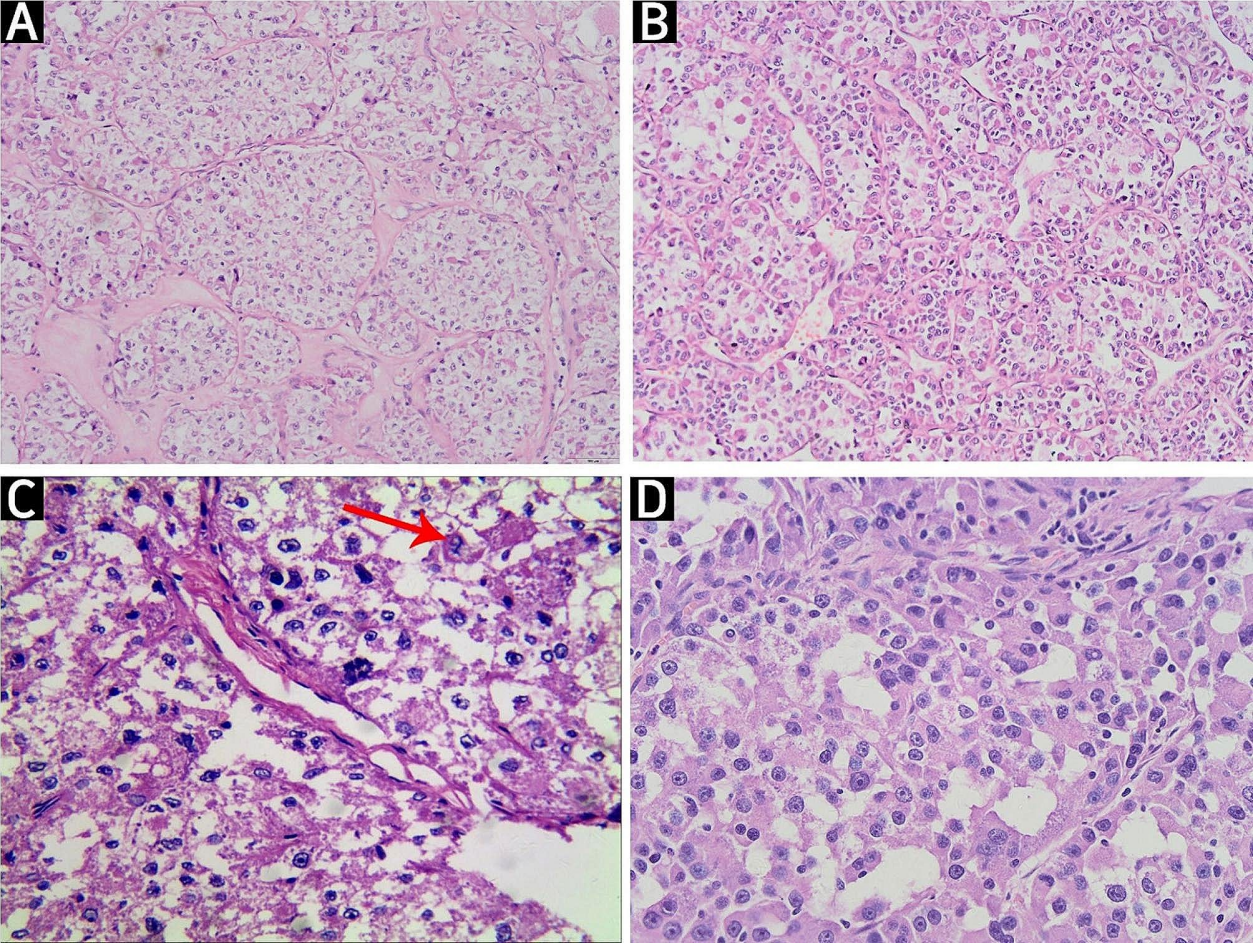
Microscopic imaging of haematoxylin and eosin (HE)-stained tumour cells. (**A**) Tumour tissue was separated into nodules of different sizes by coarse fibrous tissue. The cells are shown at 100× magnification after HE staining. (**B**) Tumour cells within nodules are arranged in a glandular vesicle shape, with rich intervesicular blood sinuses. The cells are shown at 200× magnification after HE staining. (**C**) Mitotic figures are observable at high magnification. The cells are shown at 400× magnification after HE staining. (**D**) Tumour cells of uniform size and shape (round or polygonal) are shown. Most of the cells are well defined, with abundant cytoplasm, and are translucent or eosinophilic. The cells are shown at 400× magnification after HE staining

All the tumours assessed had similar histological stages. Under low magnification (100×), tumour tissues were divided into nodules or lobes of different sizes by thick fibres (Fig. 1A). The tumour cells within the nodules were arranged as organoid or glandular vesicle-like structures with abundant blood sinuses between vesicles (Fig. 1B). The nuclei were obvious and round or ovoid in shape. A high-magnification (400×) view showed few mitotic figures (Fig. 1C). Under high magnification, the tumour cells appeared uniform in size and morphology (round or polygonal in shape). Most cells had clear cell boundaries and abundant cytoplasm that was translucent or eosinophilic (Fig. 1D).

#### Immunohistochemical findings

**Fig. 2:**
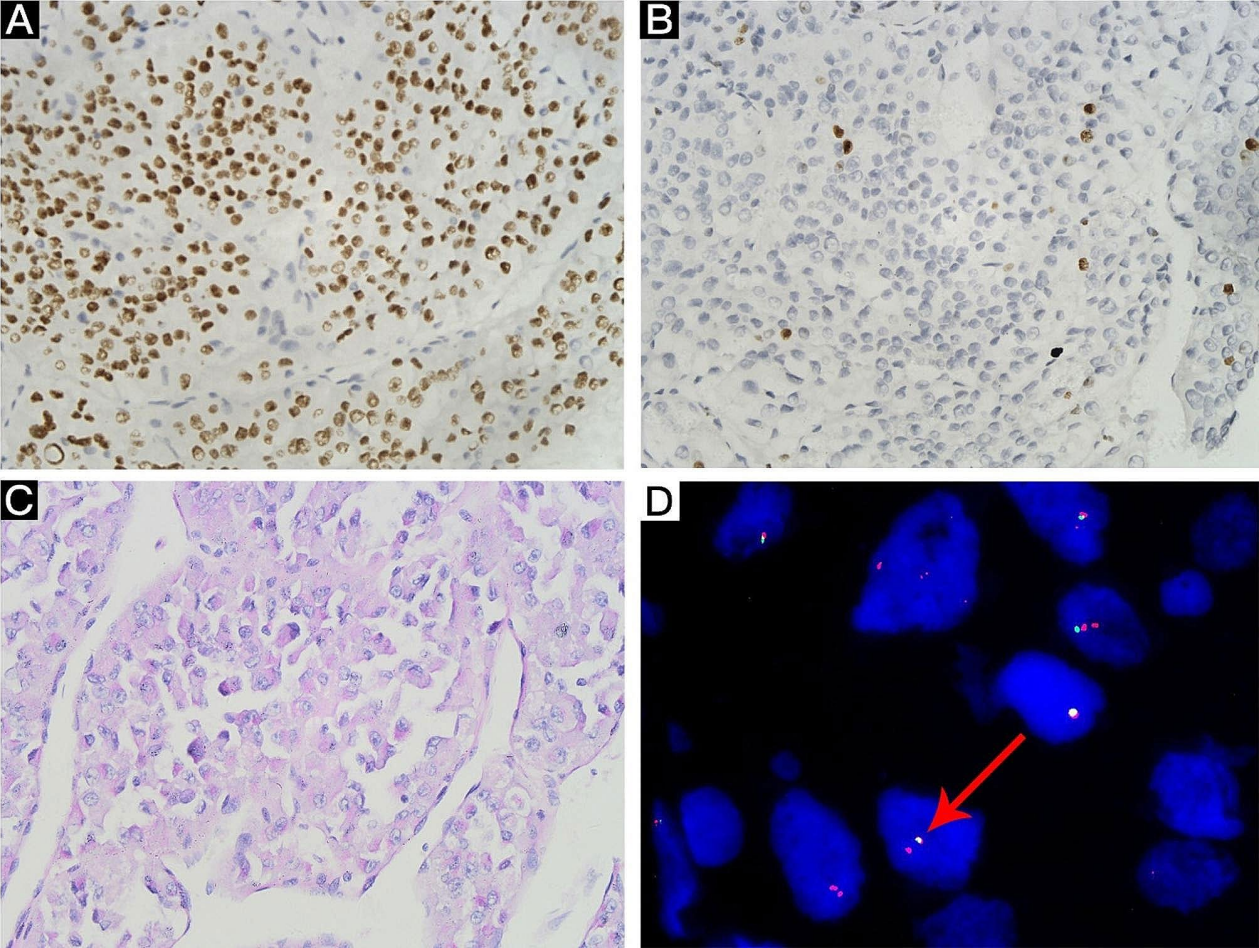
Histological and immunochemical assessment of tumour cells. (**A**) Tumour cell nuclei showing moderate-intensity expression of TFE3 (EnVision, 400× magnification). (**B**) Tumour cells with low Ki-67 value-added indices and nuclear expression are shown (EnVision, 400× magnification). (**C**) Tumour cell cytoplasm containing periodic acid-Schiff (PAS)-responsive rod-shaped crystals was observed at 400× magnification after haematoxylin and eosin (HE) staining. (**D**) *TFE3* rearrangement visualized via breakapart FISH probes is shown. One male was positive for one red band and one fusion (1R1F)

The TFE3 positivity rate was 92.3% (24/26), and the signals were localized to the nucleus (Fig. 2A). The Ki-67 value-added index ranged from 1 to 80% (Fig. 2B). Other immunohistochemical results were as follows: NSE positivity rate, 7.7% (2/26); Des positivity rate, 7.7% (2/26); CK positivity rate, 11.5% (3/26); EMA positivity rate, 7.7% (2/26); CD68 positivity rate, 11.5% (3/26); SMA positivity rate, 7.7% (2/26); CgA positivity rate, 3.8% (1/26); and S-100 positivity rate, 7.7% (2/26). The tumour cells in 20 patients were PAS positive and contained rod-shaped crystals in the cytoplasm (Fig. 2C).

#### Gene rearrangement

FISH was performed for 12 of the 26 patients, and *TFE3* gene rearrangement occurred. F indicates a normal *TFE3* red‒green (fusion) fluorescent signal and 1R indicates a red probe translocation to other chromosomes against the telomeric end, resulting in a separate red fluorescent signal. Under normal circumstances, females have two fusion signals (2 F), and males have one fusion signal (1 F), indicating that the *TFE3* gene is not breakapart. One red and two fusions were detected in females (1R2F), and one red and one fusion were detected in males (1R1F) (Fig. 2D).

#### Survival analysis

**Fig. 3:**
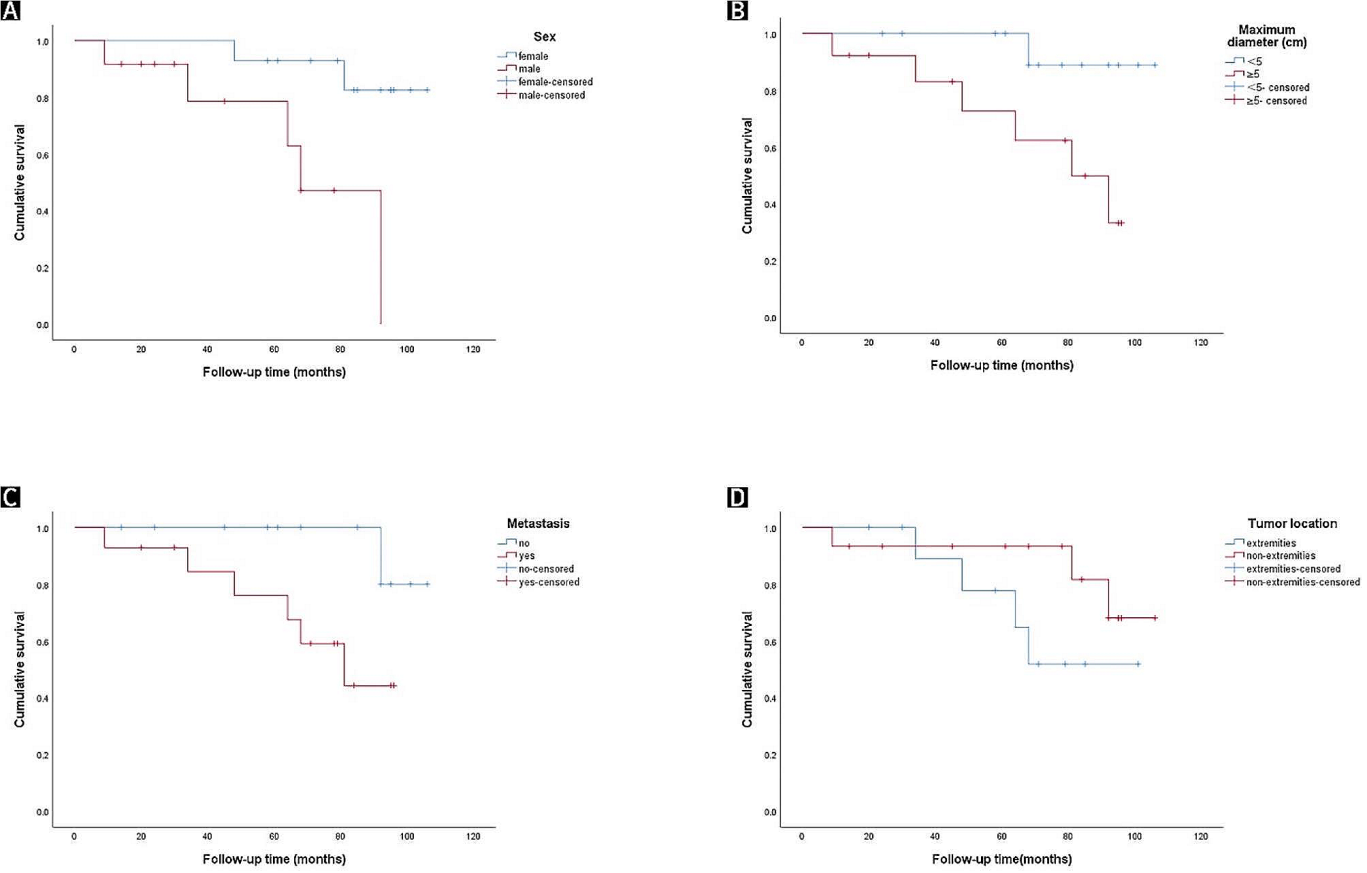
Kaplan–Meier survival curves indicating a correlation with (**A**) sex (*P* = 0.006), (**B**) maximum diameter (*P* = 0.031), and (**C**) the presence or absence of metastasis (*P* = 0.043) are shown. (**D**) Survival is shown to be unrelated to tumour location (*P* = 0.167)

KM survival analysis revealed that patient prognosis was not significantly correlated with age and tumour location (*P* > 0.05) but was significantly correlated with sex (*P* = 0.006) (Fig. 3A), tumour size (*P* = 0.031) (Fig. 3B) and metastasis (*P* = 0.043) (Fig. 3C). Cox regression analysis revealed that sex and metastasis were independent prognostic risk factors for patients with ASPS (*P* < 0.05) (Tables 2 and 3).

**Table 2:** Assignment specification of variables

Variable	Assignment specification
Sex	Female = 0, Male = 1
Tumorsize	0 = Tumour size < 5, 1 = Tumour size ≥ 5
Metastasis	No = 0, Yes = 1
Outcome	Death = 0, Survival = 1

**Table 3:** Multivariate COX regression analysis of ASPS

Variable	Beta	Standard Error	Waldχ2	Degree of Freedom	*P*	Hazard Ratio	95.0% Confidence Interval	
							Lower	Upper
Sex	-3.409	1.346	6.413	1	0.011	0.033	0.002	0.463
Tumour size	-2.346	1.327	3.127	1	0.077	0.096	0.007	1.289
Metastasis	-3.104	1.543	4.046	1	0.044	0.045	0.002	0.924

## Discussion

ASPS tends to affect adolescents, with the age of onset ranging from 15 to 35 years [3]. In our cohort, the oldest patient was aged 68 years. In adults, ASPS occurs mostly in the extremities and trunk, although there are a few reports of this disease in sites such as the meninges, lung, and breast. ASPS is mostly located in deep soft tissues and grows slowly and painlessly. Because of the rich blood supply available, tumours are prone to local recurrence and heamatological metastasis after surgery, including metastasis to the lung, kidney, brain, and gastrointestinal tract [7]. Among the 26 tumours considered in this study, 21 occurred in the deep soft tissues of the extremities and trunk.

ASPS is usually nodular in appearance and may be surrounded by a pseudocapsule. Some tumours exhibit infiltrative growth with greyish cut surfaces. Furthermore, haemorrhagic foci and necrosis are commonly observed in larger tumours. On light microscopy, ASPS has a characteristic pathological pattern of large polygonal cell nests and lobules separated by elongated fibrous septa-containing thin-walled blood vessels, with loss of intercellular cohesion in the centre of the nest. This is often accompanied by necrosis, resulting in a characteristic “glandular vesicle” pattern. The cells contain an abundant, hyaline and granular eosinophilic cytoplasm, often with eccentric, uniform round or ovoid nuclei that are vacuolated, and contain 1–2 distinct nucleoli, and rarely undergo mitosis.

Immunohistochemical findings revealed that a few tumour cells expressed S-100, NSE, Des, and epithelial markers including CK and EMA. All tumours were negative for neuroendocrine markers such as Syn, CgA, and NF. TFE3 sensitivity in diagnosing ASPS is greater than 95% [8]. However, its specificity is limited due to its expression in other tumours, such as granulosa cell tumours, neuroendocrine cell tumours, and translocation pheochromocytoma [9]. Therefore, tumour histological features and relevant immunohistochemical tests are required for a clear diagnosis.

Approximately 70–80% of the cytoplasm of ASPS cells contains glycogen that stains positive for PAS and amylase-resistant rod or bar-like structures [10]. In contrast, such material is absent in tumours such as alveolar rhabdomyosarcomas and paragangliomas. In the present study, the tumour cells in 20 patients (20/26) were positive for PAS, and the cytoplasm of these cells contained many rod-shaped or bar-shaped purplish crystals; therefore, performing adjuvant PAS staining facilitates ASPS diagnosis.

Since ASPS shares features of other tumors, considering differential diagnoses is important. ASPS is slow growing and can be asymptomatic or mildly symptomatic for long periods; therefore, prior to surgery, they are often mistaken for benign tumours. It is particularly important that ASPS be distinguished from tumours with abundant sinusoidal structures, organ-like or vesicle-like structures, or features of a vacuolated nucleus.

Paragangliomas typically occur below the diaphragm or in the bladder, with 2% of cases occurring in the neck or other rare sites. The histomorphology of this tumour is similar to that of ASPS. Notably, necrosis is typically absent in paragangliomas. Immunohistochemical findings for paragangliomas are as follows: S-100 positivity; positivity for neuroendocrine markers such as NSE, CgA and Syn but not MyoD1, Des or others; and PAS positivity without positive cytoplasmic particles.

Alveolar rhabdomyosarcoma is common in children and adolescents and occurs in the oral cavity, nose and genital tract. Immunohistochemical findings for alveolar rhabdomyosarcoma include the following: tumour cells expressing myogenic markers such as Des and MyoD1, with MyoD1 observable in the nucleus [11], which differs from the cytoplasmic positivity observed in ASPS cells.

Primary foci of metastatic clear cell renal cell carcinoma are found in the kidney. Cancer cells may be found in a glandular vesicle in a papillary or tubular pattern. The cells have abundant interstitial blood sinuses, transparent cytoplasm, and small nuclei. The morphology of renal cell carcinoma cells with the *ASPSCR1*::*TFE3* fusion is often similar to that of ASPS cells. Cancer cells may be nested with clear cell boundaries and transparent cytoplasm. TFE3 positivity is also observed; however, renal cell carcinomas with the *ASPSCR1*::*TFE3* fusion also express CK and EMA epithelial markers and are positive for CD10 and RCC.

A perivascular epithelioid cell tumour (PEComa) results in a tumour with perivascular epithelioid cell differentiation. Tumour cells may also exhibit organoid arrangement, local associations with vessel walls (usually thick-walled vessels), and pigment and smooth muscle cell marker expression, including HMB45 and Melan-A. Recent studies have revealed the presence of *TFE3* gene rearrangements in 23% of PEComas, which involve the formation of gene fusions such as *SFPQ/PSF*::*TFE3* and *DVL2*::*TFE3*. Accurate recognition of gene rearrangement is important because PEComa with *TFE3* rearrangement has different tumour clinicopathological profiles and gene expression profiles than classical PEComa that closely mirror that of ASPS. It has been suggested that the nomenclature for *TFE3* translocation PEComa should no longer be used since the pigmented Xp11 tumour nomenclature is more appropriate [12]. 

Epithelioid haemangioendothelioma (EHE) with *TFE3* rearrangement is more common in young people than in older people, and there is no significant sex difference [13]. The clinical manifestations are space-occupying lesions in the corresponding sites with associated symptoms. It is composed of streaks, nests, small clusters or single scattered epithelioid tumour cells, and the stroma is mucochondral or hyaline. Regarding immunohistochemistry, the EHE caused by *TFE3* rearrangement often shows diffuse TFE3 positivity in the nucleus, and in some cases, TFE3 is negative, but *TFE3* gene rearrangement is observed [14]. Moreover, vascular endothelial markers such as CD31, CD34, and ERG are diffusely strongly positive.

The histogenesis of ASPS is controversial, with most scholars believing that tumours are myogenic in nature. Among the 26 cases of ASPS considered, most were found in deep soft tissue skeletal muscle, a finding that is consistent with the literature. Ultrastructural observation of ASPS cells revealed needle-like or rod-like crystals structurally similar to actin filaments in the cytoplasm, an expected feature of healthy human muscle spindles. Several scholars have used reverse transcription-polymerase chain reaction (RT-PCR) to detect the expression of myoregulatory protein, a-actin, and TPM2 mRNA in ASPS tissues. In this study, myosin mRNA expression was detected in 2 patients, suggesting for the first time the myogenicity of ASPS at the molecular level [15]. 

Nonetheless, immunohistochemical results showed that muscle markers are not consistently expressed in ASPS. In fact, Des, an intermediate filament protein, is widely distributed in skeletal and smooth muscle. However, Des positivity is observed in only 40% of ASPS cases and SMA positivity is observed in only 20–30% [16]. The percentage of Des-positive ASPS cells in our study was only 7.7%. Furthermore, some ASPS expressed S-100 neural markers (7.7% positivity rate). Therefore, the tissue origin of ASPS requires further investigation [17]. 

For ASPS patients with limited tumours and no distant metastasis at the time of treatment, complete and expanded resection of the primary tumour is an important treatment method [18, 19]. Residual tumour cells at surgical margins are sources of local tumour recurrence [20]. Local recurrence is extremely rare if the primary tumour is completely resected (RO). Ogose et al. [21] reported that 36 of 38 patients with limited-stage ASPS underwent extended resection of the primary site, with 2 patients undergoing amputation. The procedures resulted in no local recurrence at the 70- month follow-up. A study from the MD Anderson Cancer Center reported that 22 patients with limited-stage ASPS underwent expanded local excision, with a median follow-up of 108 months, and that only 2 patients experienced local recurrence [22]. The significance of primary resection in the management of limited-stage ASPS is illustrated by the fact that 91.7% of the 26 patients considered in our study exhibited local control post-local extended resection of the primary ASPS site after a median follow-up duration of 69.5 months.

ASPS has a poor final prognosis despite its slow growth. The survival rate of the patients considered was 76.5% after 5 years of treatment. The 5-year survival rate of patients with metastasis was 35.3%. The survival time of adolescent patients was significantly better than that of other patients, a finding that is consistent with the literature [23, 24]. Pappo et al. [25] reported an overall survival rate of 74% at 12 years for 11 adolescent patients with alveolar soft tissue sarcoma, which was significantly better than that reported for adults with alveolar soft tissue sarcoma (34% at 10 years). [26] The effect of sex on prognosis remains controversial, with Lieberman et al. [26] concluding that sex has no significant effect on prognosis, while Daigeler et al. [27] found that men had a worse prognosis than women. This study showed that sex was associated with survival (Fig. 3A; *P* = 0.006), with female patients having higher survival rates than male patients. This finding is likely because the mean age at presentation was older in males (35 years) than in females (30 years).

Ogose et al. [21] summarized the clinical findings of 57 patients with various stages of ASPS who were treated in Japan. The authors showed that a primary tumour diameter of ≤ 5 cm was associated with 5-, 10- and 15-year survival rates of 72%, 65%, and 65%, respectively. In contrast, a primary tumour diameter of > 5 cm resulted in 5-, 10-, and 15-year survival rates of 46%, 9%, and 0%, respectively, suggesting that primary tumour size is an important prognostic indicator. Casanova et al. [23] also reported that primary tumour size was closely related to survival and the presence of distant metastasis. Pennacchioli et al. [28] reported that the larger the tumour was, the higher the index of division and the greater the risk of distant metastasis. Our study also showed that patients with tumour diameters of < 5 cm had improved survival rates compared with those with tumour diameters of ≥ 5 cm. Furthermore, the difference between the survival rates of the groups was statistically significant (Fig. 3B; *P* = 0.031). The study findings also showed that the presence or absence of metastasis was an indicator of prognosis (Fig. 3C; *P* = 0.043). However, the effects of the primary tumour site on survival are less clear. Ogose et al. [21] and Casanova et al. [23] reported no effect of the primary tumour site on patient survival, while Folpe et al. [24] suggested the opposite. The results of this study showed that the primary tumour site was not associated with survival (Fig. 3D; *P* = 0.167). Multivariate Cox regression analysis revealed that sex and metastasis were found to be independent prognostic risk factors for patients with ASPS, and male patients and patients with metastasis had a greater risk of death.

Many scholars have reported that adjuvant radiotherapy and chemotherapy are effective treatments for patients with ASPS. Nonetheless, the effect of salvage systemic therapy has not been reported to be significant [29]. A prior study showed that in 4 patients with tumours, metastasis occurred, even during radiotherapy and chemotherapy. In fact, in 1 patient who experienced recurrence at postoperative year 6, postoperative prophylactic chemotherapy and radiotherapy had no significant effect on local recurrence and metastasis. Currently, complete resection of primary site tumours is the main treatment modality for ASPS, and early detection and extensive resection are key factors affecting the effectiveness of ASPS treatment. For patients with distant metastases, surgical resection can significantly prolong asymptomatic survival. Since tumours are insensitive to chemotherapy, patients in good general condition with metastases within the range of surgical resection should have metastases surgically removed when possible.

In conclusion, sex, size of the primary tumour, and presence of distant metastases are important prognostic factors for patients with ASPS. Additionally, sex and metastasis are independent prognostic risk factors for patients with ASPS. However, the value of postoperative adjuvant radiotherapy is currently unclear. The *ASPSCR1*::*TFE3* fusion protein has been found to activate the mesenchymal to epithelial transition factor signalling pathway to promote neovascularization and cell proliferation [30]. In addition, some studies have shown that angiogenic drugs such as sunitinib are effective at treating advanced ASPS [31]. TFE3 is a relatively specific diagnostic indicator of ASPS; however, its presence must be combined with the patient’s clinical history and tissue phenotype when diagnosing ASPS. Due to the small number of patients in this study, it is necessary to expand the sample size for further study.
